# A Smart and Secure Logistics System Based on IoT and Cloud Technologies

**DOI:** 10.3390/s21062231

**Published:** 2021-03-23

**Authors:** Ilaria Sergi, Teodoro Montanaro, Fabrizio Luca Benvenuto, Luigi Patrono

**Affiliations:** 1 Department of Engineering for Innovation, Università del Salento, 73100 Lecce, Italy; ilaria.sergi@unisalento.it (I.S.); teodoro.montanaro@unisalento.it (T.M.); 2 Sinapsi S.R.L., 73100 Lecce, Italy; fabrizio.benvenuto@lesinapsi.it

**Keywords:** Azure Sphere, edge computing, end-to-end security, Internet of Things, IoT Hub, logistics, MT3620 MCU

## Abstract

Recently, one of the hottest topics in the logistics sector has been the traceability of goods and the monitoring of their condition during transportation. Perishable goods, such as fresh goods, have specifically attracted attention of the researchers that have already proposed different solutions to guarantee quality and freshness of food through the whole cold chain. In this regard, the use of Internet of Things (IoT)-enabling technologies and its specific branch called edge computing is bringing different enhancements thereby achieving easy remote and real-time monitoring of transported goods. Due to the fast changes of the requirements and the difficulties that researchers can encounter in proposing new solutions, the fast prototype approach could contribute to rapidly enhance both the research and the commercial sector. In order to make easy the fast prototyping of solutions, different platforms and tools have been proposed in the last years, however it is difficult to guarantee end-to-end security at all the levels through such platforms. For this reason, based on the experiments reported in literature and aiming at providing support for fast-prototyping, end-to-end security in the logistics sector, the current work presents a solution that demonstrates how the advantages offered by the Azure Sphere platform, a dedicated hardware (i.e., microcontroller unit, the MT3620) device and Azure Sphere Security Service can be used to realize a fast prototype to trace fresh food conditions through its transportation. The proposed solution guarantees end-to-end security and can be exploited by future similar works also in other sectors.

## 1. Introduction

In recent years, the adoption of new technologies and innovative ICT solutions are influencing almost every sector and every aspect of people’s lives. One of the sectors that has been mostly changed by such innovations is the logistics one that, thanks to the speeding up of transportation and the spread of online delivery services that is also changing the overall market. Furthermore, new contexts like Industry 4.0 have promoted the adoption of Internet of Things (IoT)-based solutions to also enhance consolidated practices [1,2,3] like the transportation of goods. In fact, in logistics, the traceability of goods and their condition monitoring during transportation have become hot issues due to the increasing interest of customers, for instance, in tracking the status of their orders. These innovative scenarios have brought us to the need of systems that, on the one hand, can plan, manage and optimize processes both along the supply chain or in complex logistics centers (such as multimodal transport centers) and, on the other hand, can achieve real-time traceability of goods or containers.

One of the characteristics that has mostly attracted the attention of logistics stakeholders involves the control of the integrity of the goods, especially in the case of perishable goods (such as fresh goods) that guided the creation of cold chain management systems able to monitor several parameters to guarantee their freshness properties. In this regard, the use of advanced technological tools and, in particular, the use of IoT-enabling technologies, represents an added value to this process thereby achieving easy remote and real-time monitoring of transported goods and providing several useful information for the management of goods (and/or containers) and the generation of warnings in case of emergencies. Such needs of monitoring are more evident in the food domain, and in the same sector the IoT has already supported different proposed solutions. In fact, for instance, the usage of radio frequency identification (RFID) and near field communication (NFC) technologies has already demonstrated how the IoT can enhance activities like the tracing and tracking of the vegetables supply chain [4,5,6,7]. Furthermore, as declared by Aung et al. [8], the monitoring of food and the traceability on the supply chain is fundamental to guarantee consumers’ safety. To this aim, different monitoring systems have already been proposed in the literature, but they become useless and, in some cases, dangerous, if there is no mechanism able to guarantee the integrity of the exchanged and stored information [9]. For example, stakeholders (including end users) would like to know that the transported goods have not suffered any thermal shock or excessive vibration during transportation, and such an information could also be valuable to give real value to the product. For this reason, another important aspect that is gaining momentum is the security in the IoT domain. In fact, even though the need of guaranteeing safety of goods and food has been recognized also by end-users, experts are aware about the highest importance of guaranteeing security of both communications and storage systems at every level and for every environment.

Another aspect that is really important for solutions specifically designed for the logistics sector relates to the timeliness needed to receive information about the actual conditions of the goods being transported (e.g., temperature, humidity, vibration, shock, etc.). In fact, the rapid perception and communication of this information can allow a fast management of emergency situations (e.g., rapid increasing of goods temperature). This requirement leads the research of effective solutions to the concept of edge computing [10]. Due to the limited resources, many early IoT devices were designed to only collect data to directly send them, without any calculation, to more complex systems responsible for the analysis. However, the ever-increasing computing power of today’s “things” has brought to the spread of the edge computing concept that allows them to perform complex calculations. Edge computing, indeed, expands cloud computing capabilities by bringing “light” services closer to the edge of the network actually supporting a variety of new services and applications, in particular when there are serious constraints in terms of delay. In fact, by processing data closer to the source and prioritizing traffic, edge computing reduces the amount of data flowing into and out of the main network, thereby reducing latency and increasing overall speed, and providing highly responsive and context-aware real-time insight and immersive experience. Cloud service giants are also making enormous investments in this area to foster the research of innovative solutions able to guarantee all the discussed properties and, at the same time, able to support developers and practitioners at all the level of the implementation of a solution. Due to the fast changes of the requirements and the difficulties that, consequently, researchers can encounter in proposing new solutions, the fast prototype approach could contribute to rapidly enhance both the research and the commercial sector. It represents the possibility of quickly create a prototype of a solution before the real implementation of the final product to facilitate the validation of the concept and the introduction of improving ideas.

As a summary, the logistics sector needs comprehensive solutions to support researchers in realizing fast-prototyping solutions to be deployed at the edge level and that can grant security in communications and storage at every level.

Recently, Microsoft launched “Azure Sphere”, an edge computing platform used to build low-cost connected devices that can be remotely managed and monitored. It consists of hardware (i.e., s microcontroller unit, the MT3620 [11]), software components (based on a custom advanced Linux operating system) and a security service called “Azure Sphere Security Service” [11]. Microsoft Azure Sphere is a new solution for creating highly secure, internet-connected MCU devices. It includes multiple components that can work together to protect and power devices at the intelligent edge. As discussed in the following sections, its main advantages with respect to other existing solutions used in works present in literature regard the end-to-end security that guarantees data communications and storage protection against malicious situations.

By exploiting an integrated solution including Azure Sphere certified devices and Azure Cloud services, this paper proposes an intelligent platform to support fast prototyping of cold chain management systems at the edge level with specific focus on guaranteeing end-to-end security. In particular, the proposed work is focused on real-time data collection, local data processing and data sending to a secure cloud platform, while leaving further data processing and presentation for future development. This process can be further enhanced in future works by leveraging other services provided by Microsoft Azure (for example, the Azure features for artificial intelligence) or using third-party services or ad-hoc developed services. The feasibility of the proposed solution has been validated through a real proof-of-concept application.

The paper is structured as follows: In Section 2, the state of the art of innovative solutions proposed in the literature for logistics management and, in particular, for the monitoring of goods using IoT solutions is presented. In Section 3, the main system requirements and constraints are analyzed, and in Section 4 the materials and methods used to realize the proposed solution are presented. The proposed architecture is introduced in Section 5 and each system component is described in detail. The test procedures to validate the whole system from a functional point of view and main results are presented in Section 6. In Section 7, a brief discussion about main open issues as well as a comparison of the proposed solution with similar ones analyzed in the state of the art are reported. Finally, conclusions are drawn in Section 8.

## 2. State of the Art

As declared by Zhou et al. [12] in the past few years, transport services have attracted the attention of many researchers and practitioners who have investigated methods and solutions to improve the logistics obtaining more efficient delivery of goods. This scientists’ effort has been especially fed by the advent of the IoT concept that is affecting substantially the logistics [13]. Indeed, as highlighted in the investigation performed by Ivankova et al. [14], different works are proposed in the literature with the aim of highlighting the positive effects of IoT in logistics. Various proposals, in fact, can be identified in the literature proposing frameworks [15] and management systems [16,17,18] to enhance logistics through IoT, for instance, in the maritime industry [19] or the garbage industry [20,21].

However, there is a specific aspect related to transport management that has been mainly influenced by the advent of the IoT: the traceability of goods. This paper focuses on this aspect by proposing a solution that highlights the benefits that IoT, and its specific branch called edge computing, can bring in the goods traceability domain by exploiting innovative solutions for fast prototyping and specifically concentrating on security requirements at the edge level.

Unfortunately, to the best of our knowledge there exist only a few works [22,23] focused on fast prototyping solutions for goods traceability through edge IoT devices specifically concentrated on security, consequently, the following paragraphs will analyze existing works by dividing them into three main groups: one that presents systems to monitor goods through edge devices during transportation, another one that discusses existing artefacts that propose systems able to guarantee security for collected and transmitted data in goods traceability at the edge level and the last one focused on the specific topic of the paper to present the few works focused on all the topics of this paper (i.e., fast prototyping, goods traceability, and security at edge level).

### 2.1. Goods Traceability through Edge Devices

Different works have been proposed in literature with the aim of enhancing good traceability through edge devices. Chang et al. [24], for instance, presented the design and implementation of an intelligent “tape” to monitor the transport procedures of specific products’ shipments like high-price and fragile cargo. To avoid the possibility of abnormal charging and discharging, a thin film piezoresistive pressure sensor is installed in proposed iTape and then, through piezoresistive sensors, actually acting as edge devices, it communicates to a central system the anomaly and create warnings. The communications are established and guaranteed by an 8051-like microcontroller unit (MCU) by Intel (Santa Clara, CA, USA). Differently from our solution the proposed system does not exploit fast prototyping solutions and do not specify if any security measure is applied. In addition, the used MCU does not provide any hardware functionality for end-to-end security.

Chen et al. [25] proposed a cold chain system to monitor perishable food products during storage, transport and sale for guaranteeing the freshness of monitored goods. The paper is mainly a concept presentation concentrated on the specific technology used for the observations, but the proposed idea is actually able to detect the temperature of the transported goods like the system presented in this paper. The paper does not treat any implementation detail and, consequently, its similarities with our work regards only the concept.

The approach followed by Muñoz-Gea et al. [6], instead, is more similar to the one adopted in our paper, however those authors mainly focus their attention on the infrastructure used to establish a network of devices using the EPCIS standard. In fact, they propose a traceability system that exploit device installed at the edge level (even though the edge concept was not yet completely present in literature at the time of that paper writing) through the FreePastry software to demonstrate the feasibility of the system and highlighting some security problems that were solved within the work. Specifically, authors highlight how the used FreePastry software and their additions can guarantee different security properties. However, no hardware device is used within the proposal and, this characteristic mainly differentiate their work from ours. In fact, the security measures that our work adopt at the hardware level are only adopted at a higher level, leaving some security issues unresolved. In addition, the used technologies are not fast prototyping solutions.

Qian et al. [7] present another traceability system that, by incorporating 2D barcode and RFID technology, allows them to trace wheat flour. The system exploits edge devices to monitor the products and was evaluated using a contrast experiment divided into five parts, including raw material data recording, processing data recording, pack-age data recording, logistics data recording and traceability query. The results demonstrated the efficacy of IoT platform in all these processes and specifically in the traceability one. This work is another example of existing solutions that try to adopt the same approach used in our paper. In the proposed work, identification information are stored on “Alien Technology ALN-9654 G Inlay” Tags and then read by a “Sense Technology S1853” reader connected to a computer. Then, the information is sent to a web database through a GPRS connection. It seems again that no fast-prototyping platforms are used and the security measures are not specified. In addition, the specified hardware does not provide any end-to-end security measure.

Wanganoo et al. [26] propose an integrated conceptual framework to support real-time visibility and decision-making in the monitoring of temperature environment storing goods in the cold supply chain. The proposed solution was not validated through a real use case, but the idea of attaching an edge sensor on the pallet on which the goods are stored is a good way of tracing the goods conditions also in the transportation process. The work does not provide information about security measures and fast prototyping tools that could be adopted in future implementation of the concept.

Chen et al. [27] presented a system to monitor and control petrol, oil and lubricatant (POL) transportation. Specifically, apart from the traceability of the GPS position of the truck, the system is able to monitor also the temperature in addition to attempted thefts. In case of strange temperature values of the transported fuel, an alarm is generated to alert the driver and promptly intervene. The prototypal solution does not discuss details about the used hardware and software and, consequently, authors do not explain neither how the security is guaranteed throughout the whole data chain nor if any fast prototyping tool was used.

### 2.2. Security in Goods Traceability at the Edge Level

In this section two main types of related works are presented. First works that are concentrated on security algorithms to be used in the edge are presented, while the second part reports the solutions that exploit the so-called distributed ledger technology (DLT) to guarantee the integrity of information related to transporting goods. Unfortunately, neither of these types of solutions is adequate for fast prototyping.

Zhao et al. [28] presented an agricultural traceability system to guarantee the security of information acquired at the edge level. The proposed solution exploits specific IoT tags combined with an encryption mechanism able to certify the trace of a product, i.e., the history, application or location of that product. The proposed encryption mechanism hardly fit the fast prototyping requirement considered in our work.

Belu et al. [29] propose a specialized IoT interface that ensures the protection and integrity of the data transmitted between endpoints. The protection is provided by an encryption algorithm based on two keys stored at the source and the recipient and on a very simple but efficient coding/decoding scheme. Also in this case, the proposed encryption mechanism hardly meet the fast prototyping requirement.

Masood at al. [30] exploit IoT technologies communicating over 5G networks and cloud computing to propose an algorithm to improve security of logistics and transportation. In the proposed concept, encryption-decryption is applied to encrypt data before sending it, so that the information being exchanged cannot be attacked during the process. Differently from our need of using fast prototyping solutions, the presented solution is a proposal that is not yet ready for use in fast developments, in fact it is expected that developers will implement it by themselves.

Gai et al. [31] propose an edge scheme based on the blockchain technology to guarantee security in storing and communicate data to facilitate food safety management. The proposed solution aims at collecting data about the food supply chain through edge devices to then upload them to the blockchain for record-keeping. In this way security is ensured through the blockchain technology. The proposed architecture was not yet implemented in any prototype and seems not to be adequate for fast prototyping mainly because of the lack of feasibility verification and the lack of available documentation to use the proposed solution.

Li et al. [32] present a traceability system model based on the Blockchain technologies. Blockchain provides a new tool for tracing business and guarantees the openness and transparency of the ledger by also avoiding the possibility that the stakeholders can tamper with the ledgers. Also in this case the proposed solution is not yet ready for fast prototyping.

Madumidha et al. [33] exploit a fully decentralized blockchain based traceability system to be integrated with IoT devices from provider to consumer to brings transparency in the network. Authors introduced the “provider-consumer network” concept, a theoretical end to end food traceability application to create a distributed ledger that is accessible by all users in the network. This solution is more integrable than the previous ones, however, the usage of the blockchain is not yet ready for fast prototyping requirements.

### 2.3. Exploitation of Fast Prototyping Solutions for Guaranteeing Security in Goods Traceability at Edge Level

As highlighted by the works presented in the previous sections, the security strategies are mainly adopted at the application layer, without caring about the possible attacks that can be exploited in the edge. In addition, if any work proposes a solution to guarantee security at the edge level, the solution is not appropriate for fast prototyping. However, a few existing works propose solutions to be exploited in the development of fast prototyping solutions for guaranteeing security in goods traceability at the edge level.

For instance, Wrona et al. [22] present a proof-of-concept configuration to assure security at the edge level in logistics chain monitoring. Their approach is similar to the one used within the current paper, in fact they exploit fast prototyping tools to develop a prototype of the proposed system, but the security algorithm is implemented within the paper and it seems it is not appropriate for the usage in fast prototyping.

Furthermore, Fedchenkov et al. [23] exploit different fast prototyping solutions like the FIWARE Orion Context Broker to trace waste throughout the whole chain (from citizens’ garbage containers to recycling factories). The proposed architecture is in a very prototypal form but, considering the infrastructure created and supported by FIWARE that is connectable to the Orion Context Broker, it could be used in future works to realize a system similar to the one described in this paper.

To summarize, to the best of our knowledge, there exist only a few works focused on fast prototyping solutions for goods traceability through edge IoT devices specifically concentrated on security, but their solutions are not anyway usable by developers that want to quickly develop prototypes for their purposes. Consequently, the present paper proposes a solution that could support the fast prototyping of transport logistics’ works that want to guarantee end-to-end security also at the edge level. The proposed solution exploits the advantages offered by the Azure Sphere platform, a dedicated hardware (i.e., microcontroller unit, the MT3620 [11]) device and Azure Sphere Security Service.

## 3. Main Requirements and Constraints

Starting from the presented related works that propose three main groups of existing solutions to track and monitor food conditions during its transportation, the following paragraph presents the main requirements extracted from literature that community would need in designing and implementing cold chain management system. These requirements will guide both the design and the development of an IoT device to be installed in the truck to monitor the selected parameters.

The section is divided into two parts: the first one discusses the requirements of a cold chain management system needed to support the monitoring of perishable goods, while the second part presents the needs of researchers and practitioners acting in the sector and aiming at developing a cold chain management system.

The exposed requirements will guide the design of a system architecture to support fast prototyping in goods transportation at the edge level specifically focused on security.

### 3.1. Cold Chain Requirements

A cold chain management system is usually adopted to monitor several parameters related to fresh goods (e.g., meat, fish, fruit, etc.) transported in trucks or containers or held in storage rooms. Then, usually, the collected data are processed by specific business logic modules based on artificial intelligence techniques to both provide statistical information (e.g., the average value of the temperature registered during a day) and generate warnings to authorized users. The correct collection and processing of the stored information can ensure the correct preservation of the state of transported goods.

In details, the following list summarizes all the requirements that the proposed system should include:

- monitor the container temperature; the temperature can fluctuate among −10 °C and +50 °C;
- monitor the humidity of the container; it can fluctuate between 0% and 100%;
- monitor the acceleration of the container; it can fluctuate between 0 km/h and 200 km/h;
- monitor the brightness inside the container (useful for detecting the opening/closing of the container doors);
- guarantee the collection of data also in case of connection absence;
- include a buffering mechanism for asynchronously sending data to the backend according to established rules;
- ensure end-to-end security to protect IoT devices and data;
- allow the dynamic configuration of the rules used to send data for the readaptation of the solution to the specific scenario (e.g., transportation of fish is different from the transportation of meat).

### 3.2. Cold Chain Developments’ Requirements

As already discussed in the Introduction, the transportation of goods has attracted the attention of different stakeholders in the last years and the advent of new technologies like the IoT has promoted the development of new solutions and innovations in the logistics sectors.

Due to the fast evolution of such sector and the continuous introduction of new tools and platforms, researchers working in the sector need instruments to follow the evolving trends and rapidly construct hands-on and demonstrators to validate new ideas and innovations. To this aim the fast-prototyping approach has become to help them in their daily activities and, in a short time, it become an essential requirement for the research lead in the logistics sector.

Nonetheless, the fast development of solutions cannot obscure the essential requirement of security in both communications and storage of collected data. In fact, the IoT scenario is full of examples of security leaks exploited by malicious attacks to interfere in daily activities and causing several problems.

In addition, in the last decades, the IoT is fostering the adoption of innovative habits in almost every sector. The IoT allows, in fact, to connect common devices (e.g., a lamp) to other devices and systems with the aim of offering and exploiting services that can significantly enhance existing solutions. In this scenario, the “cloud computing” paradigm has become very familiar in our daily lives. It represents the possibility of simple devices to leverage on complex infrastructure physically located in another place to collect, store and process data from the field without the necessity of investing in local resources. In fact, a smart thermometer leveraging on cloud computing is able to create warnings for emergency situations without any necessity of high computing resources: it sends collected data to a server that elaborates it and, in case, generate a warning sent back to the smart thermometer.

Such kind of architecture has been used since some troubles became evident. In fact, as highlighted by Puliafito et al. [10], the cloud computing paradigm can be used up to moment in which, for instance, latency or bandwidth consumption become important requirements (e.g., in a fire detection system). In these cases, the edge and the fog computing paradigms are becoming very popular.

**Figure 1:**
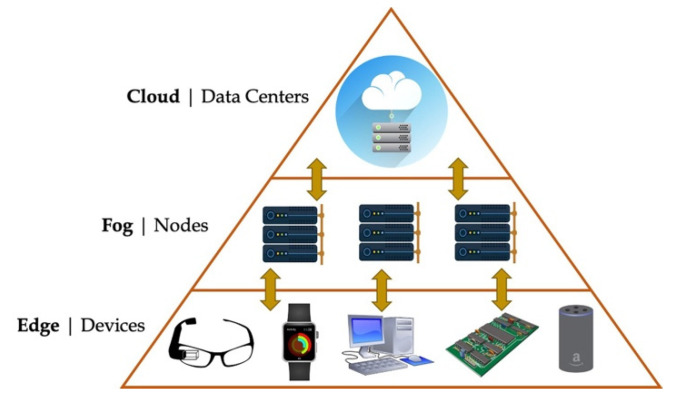
Typical edge computing architecture.

As depicted by Figure 1, the edge devices simply represent nodes that are deployed in a distributed manner and are usually placed near the gathering places of people [34,35]. They have the characteristics of limited resources that can both collect data from the field and perform simple operations at the edge of the network. They, then, send the collected, and in case elaborated information to the cloud through the Fog nodes. The fog nodes are not essential, and in fact in some cases they are only network routers or hub (without any intelligence). However, they represent a middle step in which an initial elaboration of the data can be performed before sending requests to the cloud. Fog nodes are usually devices that are more intelligent, mainly in terms of available resources, than the edge one, but less intelligent than the cloud one.

The usage of edge computing brings different advantages such as the possibility of collecting information also in case of connection absence or elaborate simple data to, for instance, warn about a dangerous situation. For this reason, edge computing has been selected for the purpose of this paper. Its features are, in fact, in line with the presented requirements and can be also exploited by other researchers and developers that will look for a fast prototyping solution for the goods traceability.

Nonetheless, there is an aspect that is usually neglected at the edge level: as declared by Zeyu et al. [34], due to the limited available resources, many traditional security mechanisms are difficult to be fully applied on edge devices. Therefore, edge nodes are easily invaded by attackers and, due to the sensitivity of the data that they could collect, the security of edge nodes is crucial and challenging [34].

To this aim, Hunt et al. [36] identifies seven properties required in all highly secure devices that are summarized in the following list:

- Hardware-based root of trust. It ensures security at hardware level through unforgeable cryptographic keys generated and protected by hardware.
- Small trusted computing base. It represents the division of software into self-protecting layers through private keys stored in a hardware-protected vault.
- Defense in Depth. It includes the provision of countermeasures to mitigate the consequences of a successful attack on any vector.
- Compartmentalization. It represents hardware-enforced barriers between software components to prevent breaches in one from propagating to others.
- Certificate-based authentication. It identifies the measures provided to prove device identity and authenticity proven by signed certificate.
- Renewable security. It represents the renewal of both software and keys/tokens to guarantee forwarding to secure states and revoking of compromised assets for known vulnerabilities or security breaches.
- Failure reporting. It identifies the capacity of a system to recognize failures and problems and report them to failure analysis system.

## 4. Materials and Methods

To address all the discussed requirements, Microsoft has proposed the Azure Sphere platform that is an end-to-end solution for securing microcontroller (MCU)-powered devices [11]. As reported by authors, the Microsoft Azure Sphere platform comprises three main components: an Azure Sphere class of MCUs, an Azure Sphere OS, and a cloud- based Azure Sphere Security Service.

This section compares the solutions and platforms exploited in the state of the art with such innovative solution proposed by Microsoft to finally select the best combination to be used in our work.

Table 1, Table 2 and Table 3 summarize the main requirements discussed in the previous section and analyze how the related works presented in Section 2 satisfy them.

To the best of our knowledge, as depicted in the tables, none of the existing systems satisfies all the requirements. For this reason the Microsoft Azure Sphere platform has been selected for the proposed solution and its services will be exploited as fast prototyping technologies within this paper and the use case will demonstrate how they can actually enhance the development of goods traceability solutions.

### Hardware Selection and Setting

**Figure 2:**
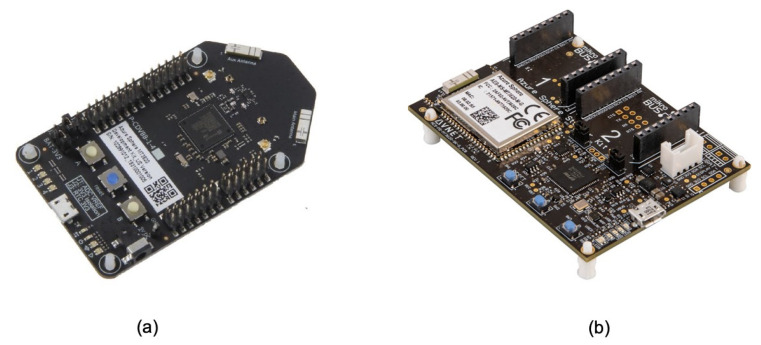
Azure Sphere MT3620 development kit by Seeed Studio (**a**), Azure Sphere MT3620 starter kit by Avnet (**b**).

The hardware used to collect data from the field is based on two different MT3620 development kits:

- the Azure Sphere MT3620 development kit by Seeed Studio [37] (Figure 2a)
- the Azure Sphere MT3620 starter kit by Avnet [38] (Figure 2b).

These two kits are designed to support rapid prototyping and enable developers to experience Azure Sphere technology. At this stage, a prototype solution has been created and two development kits have been used to meet the requirements related to monitoring parameters. Azure Sphere is a new technology. There are actually only a few certified Azure Sphere boards on the market, and only a few external sensors and related libraries have been formally tested on these commercial boards. In future work, we are working on a single board based on the MT3620 MCU, which can provide all required parameters and combine the on-board sensors with external sensors.

In Table 4, the main technical specifications of the above kits are reported. For the Avnet starter kit, only data related to the on-board sensors are sent to the IoT Hub, especially:

- 3-axis accelerometer and gyroscope
- barometric pressure

In addition, the Avnet board also provides an unreliable temperature value (because it comes from the sensor embedded in the inertial module), the elevation (absolute altitude) derived from the barometric pressure and the Wi-Fi signal strength, which is useful for detecting whether there is a Wi-Fi signal, and establish appropriate mechanisms to avoid data loss.

**Figure 3:**
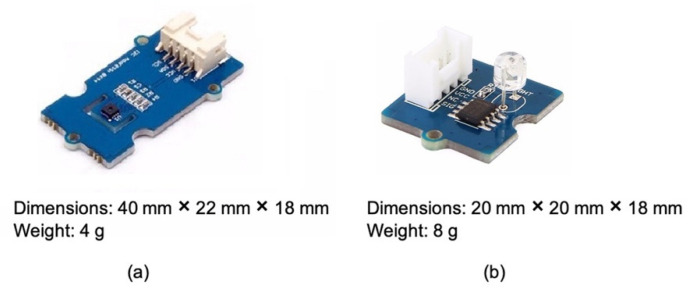
Grove Temperature & Humidity Sensor (SHT31) (**a**), Grove Light Sensor v1.2 (**b**).

With the Seeed Studio development kit, two external sensors have been used, the Grove Temperature & Humidity Sensor (SHT31) [39] (Figure 3a) and the Grove Light Sensor v1.2 [40] (Figure 3b), which can provide the following parameters:

- temperature and humidity
- ambient light.

The Grove Temperature & Humidity Sensor (SHT31) is a highly reliable, accurate, fast-response integrated temperature and humidity sensor. The temperature sensor has a range of −40 °C ÷ 125 °C with an accuracy of ±0.3 °C, while the humidity sensor has a range of 0% ÷ 100% (relative humidity) with an accuracy of ±2%.

The Grove Light Sensor v1.2 by Seeed Studio (Nanshan, Shenzhen, China) is an analog module that integrates LS06-S photoresistor (a highly sensitive and reliable photodiode) to detect the light intensity in the environment. In more detail, the resistance of the photoresistor decreases as the light intensity increases. The onboard dual OpAmp LM358 chip by Seeed Studio (Nanshan, Shenzhen, China) generates a voltage corresponding to the light intensity (i.e., according to the resistance value). Finally, the output signal is an analog value that increases with increasing brightness. Its response time is about 20–30 milliseconds.

**Figure 4:**
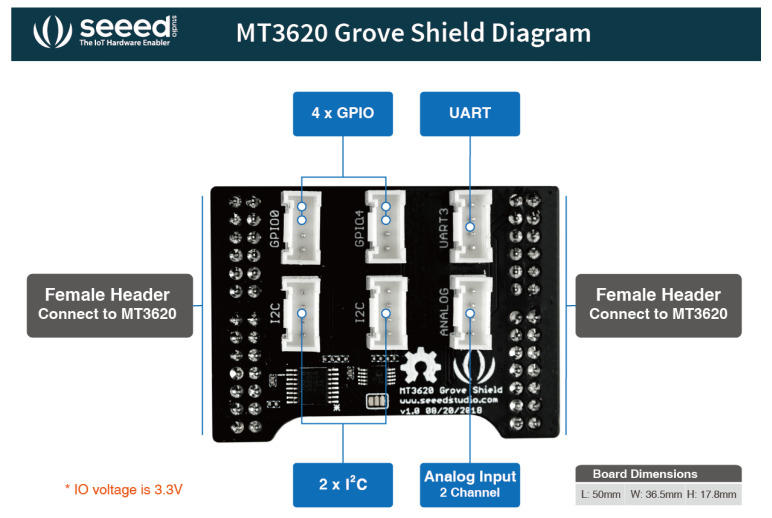
MT3620 Grove Shield by Seeed Studio.

These two sensors are connected to the Seeed Studio board through the MT3620 Grove Shield by Seeed Studio, Nanshan, Shenzhen, China (Figure 4), which is an additional breakout board designed for MT3620 Development kit. It provides six Grove connectors: one UART, two I^2^C, one analog, four GPIO. The Grove Temperature & Humidity Sensor can be connected to the analog connector, and the Grove light sensor can be connected to the Grove Shield I^2^C connector.

Once the hardware used to realize the Smart Box has been selected, the boards have been plugged into the pc via USB and configured by installing the Microsoft Azure Sphere SDK provided by Microsoft (https://docs.microsoft.com/it-it/azure-sphere/install/install-sdk?pivots=visual-studio#install-the-azure-sphere-sd, accessed on 22 March 2021).

Next, the following steps have been performed:

- Device claim. Each device has been claimed into an Azure Sphere tenant. The tenant represents an organization. It is a dedicated Azure Active Directory service instance received by the organization when registering for a Microsoft Cloud service. This operation binds the specific device to the tenant immutably, thereby associating the immutable device ID with the tenant. The Azure Sphere Security Service uses the device ID to authenticate the device.
- Network configuration. The Wi-Fi has been configured on each Azure Sphere device. To configure the Wi-Fi, some instructions have been typed from the command prompt to set the network ssid and password. The main commands are reported in Table 5.
- Setup of the Azure IoT Hub. An Azure IoT Hub has been set to work with the specific tenant. When the boards go online for the first time, they are authenticated by the tenant authentication certificate. All steps to generate certificates and associate them to the tenant are described in [41].

It is important to note that during this prototype stage of the system, a Wi-Fi connection is used, and the Azure Sphere boards are powered through the PC’s USB port. Obviously, in the future stage, IoT devices must be placed in mobile containers, then 4G connectivity must be provided, and IoT devices must be battery powered.

After configuring the boards, the Visual Studio development environment [42] has been used to develop the firmware of each board in C language. In particular, the extension for Azure Sphere [43] has been exploited. Table 6 summarizes the main files and their main implemented functions.

In order to minimize data loss, the system checks the Wi-Fi connection before sending each data. If not available, the data are stored in a circular buffer on the board. When the Wi-Fi connection is available again, all buffered data will be sent to the IoT Hub. Due to the memory limitation of each board, a fixed size buffer has been created. The buffer is configurable, and the maximum number of elements to be stored can be configured through the backend. Since the buffer is circular, if it is full, the new data will replace the old data.

## 5. Proposed System Architecture

**Figure 5:**
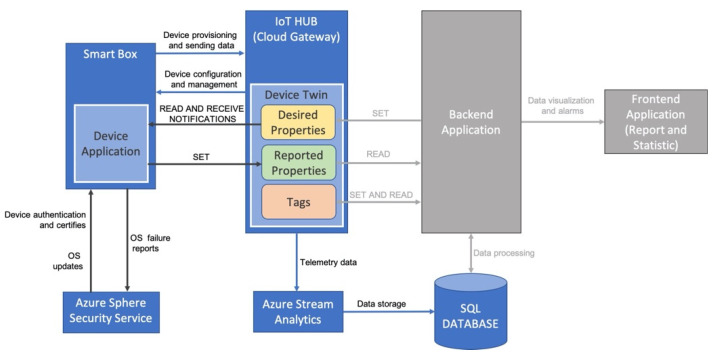
Block diagram of the proposed system architecture.

Based on the discussed system requirements and technological overview, the architecture reported in Figure 5 has been designed.

It is based on the Azure IoT Reference Architecture and is made up of different blocks. The Smart Box is responsible for collecting data from the field and is based on the Microsoft Azure Sphere MCU MT3620. It can communicate with the Azure Sphere Security Service for certificate-based authentication, failure reporting, and over-the-air software updates. The MCU MT3620 device will be connected through a Wi-Fi connection to the IoT Hub.

The Azure IoT Hub provides a secure two-way communication channel between the IoT device and the Backend Application. In addition, it plays a fundamental role in the proposed architecture as it acts as a bridge between the MT3620 device and the backend application. It supports device-to-cloud communication to collect real-time status of IoT devices, and also supports cloud-to-device commands and notifications to update strategies stored in IoT devices to collect sensor data. With the help of an authentication process (based on tokens or certificates), each device can be safely connected to the IoT Hub and managed in the same secure way. When the device is started for the first time, the IoT Hub Device Provisioning Service (DPS) will automatically provision the device to the correct IoT Hub (https://docs.microsoft.com/it-it/azure/iot-dps/about-iot-dps, accessed on 22 March 2021). Furthermore, another important feature offered by the IoT Hub is the management of device twins, which is used in the proposed system to meet the configuration requirement of remote IoT devices. Device twins are JSON documents that store device status information (including metadata, configuration, and conditions), and devices and backends can use them to synchronize device conditions and configurations. The device twin is linked to the corresponding device identity, so it will be implicitly created and deleted when the device identity is created or deleted in the IoT Hub.

The JSON file describing a device twin contains:

- Tags. The part of the JSON document that the backend can read and write. The label is not visible to applications running on IoT devices.
- Desired properties. They are used in conjunction with reported properties to synchronize device configurations or conditions. The backend application can set the desired properties, and the application running on the IoT device can read them. If the required properties change, the application on the device can also receive notifications.
- Reported properties. They are used in conjunction with desired properties to synchronize device configuration or conditions. The application running on the IoT device can set the reported properties, and the backend can read these properties.
- Device identity property. The root of the JSON document of the device twin contains the read-only properties of the corresponding device identity stored in the identity registry. In Section 7 an example of device twin is reported.

The data received by the IoT Hub are stored in a *SQL database* using a Stream Analytics Job. These data will contain as mandatory fields the following information:

- timestamp
- values detected by sensors.

More fields can be introduced by developers. The *Azure Stream Analytics* is a very useful tool that can be used to analyze real-time telemetry data streams from IoT devices because it can analyze and process large amounts of fast streaming data from multiple sources at the same time. The configured Azure Stream Analytics Job includes:

- an input, represented by the IoT Hub
- a query, used to manage (filter, sort, aggregate, join) the data stream.
- one output represented by the data storage in the SQL database. There may be multiple outputs. For example, the data can be stored in a database or sent to other Azure services (for example, sent to the Power BI report dashboard for real-time dashboard processing).

Finally, the *Backend* and *Frontend* applications will use the data stored in the database for further processing and presentation, respectively.

In this prototypal version of the system, with respect to Figure 5, only the blue blocks have been fully implemented. The gray blocks, in fact, will be implemented in future works.

## 6. Results

In the validation phase, the dataflow usually generated by a cold chain management system has been emulated in a laboratory environment to verify the feasibility of the proposed system. The following paragraphs report the details of the configuration of the boards for the performed functional validation and show the results obtained when the realized system performs its main actions, which are:

- reading data from sensors
- data control
- sending data to the IoT Hub.

**Figure 6:**
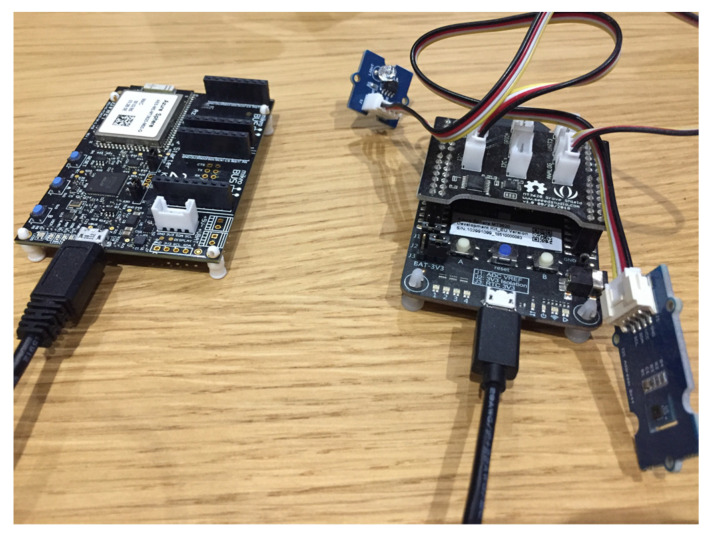
Connection of the two configured Azure Sphere boards.

For each action, appropriate screenshots will show the resulting configuration and the corresponding behavior of the system. As depicted by Figure 6 that shows the Azure Sphere boards configured with all the required sensors, all the sensors described in Section 5 and Section 6 were installed and used within the experiment.

### 6.1. Reading Data from Sensors

After configuring the boards according to the specifications defined in Section 5 and Section 6, these boards will immediately start communicating with the IoT Hub to transmit the data detected by the sensors after the automatic connection phase.

**Figure 7:**
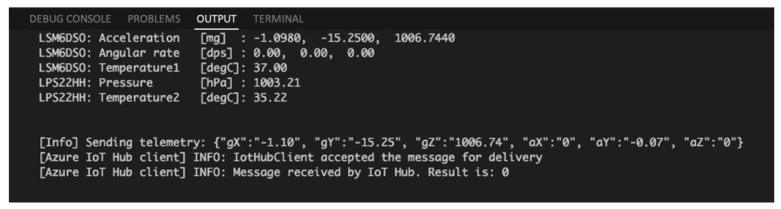
Device output in Visual Studio.

Figure 7 shows a screenshot taken from a window that is related to the Device Output console in Visual Studio, where telemetry data can be monitored before they are transmitted to the IoT Hub.

### 6.2. Data Control

**Figure 8:**
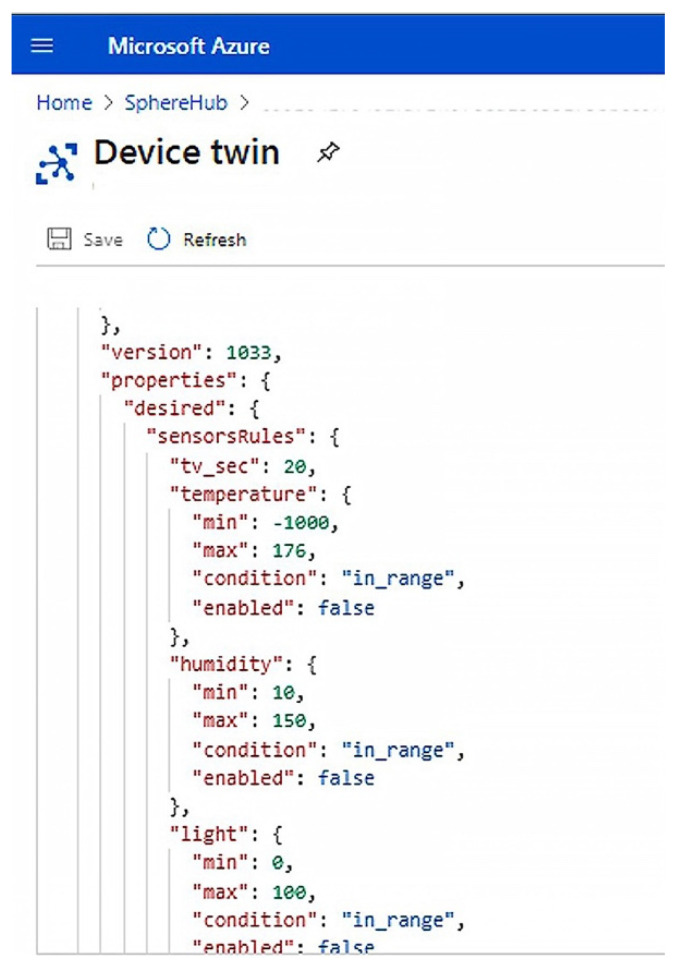
Device twin.

The control of the values coming from sensors is entrusted to the device twin, in which the rules for each sensor have been mapped. In Figure 8, an example of device twin is reported. In detail, it shows how to modify the device twin in the IoT Hub portal to define the minimum and maximum thresholds for each sensor or disable the data sending from one or more sensors.

In the device twin used for validation, the minimum and maximum values have been set for each sensor, and data transmission is enabled only if the value detected by the sensor exceeds the range expressed by the minimum and maximum values. This is obtained through the key/value pairs “condition”: “in_range” and “enabled”: “false”. Table 7 lists all the parameters defined to manage the data transmission from the IoT devices whereas Table 8 specifies the meaning of each option for the “condition” parameter. These parameters can be used in device twin to set data transmission rules.

### 6.3. Sending Data to the IoT Hub

**Figure 9:**
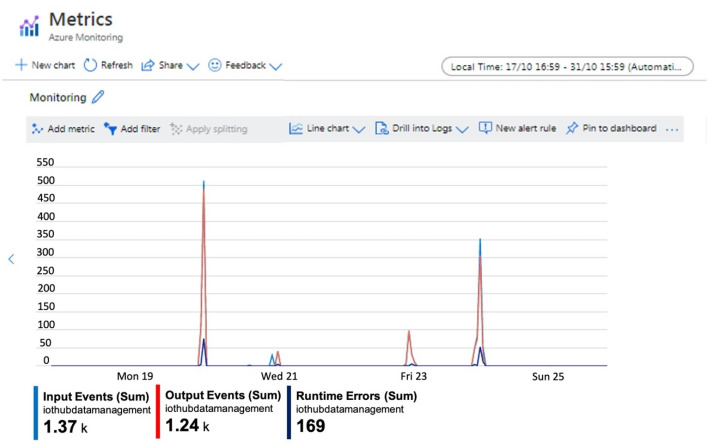
Azure Stream Analytics Job metrics.

The Azure portal allows to visualize key performance indicators useful to monitor queries and handle performance issues and troubleshoot them. An example of the metrics set to monitor the developed system is reported in Figure 9 where some metrics have been selected for flow analysis. In detail, the metric “Input Events” indicates the number of records deserialized by input event. This count does not include incoming events that generated deserialization errors. The metric “Output Events” indicates the number of events sent by the Stream Analytics job to the output target. Finally, the metric “Runtime Errors” indicates the total number of errors related to query processing.

**Figure 10:**
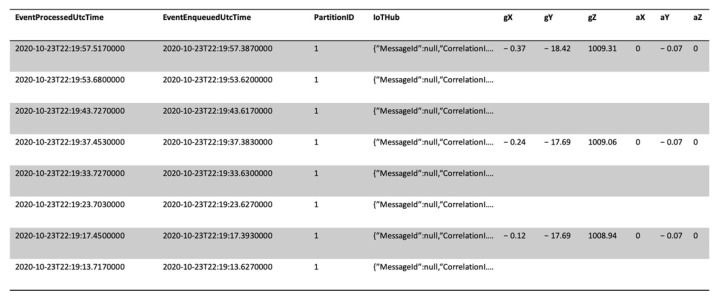
Query result on the database.

The defined job writes collected data into the connected SQL database. The table in the database is populated correctly in real time, as showed in Figure 10. Therefore, the stored data can be made available to Azure services or external services.

**Figure 11:**
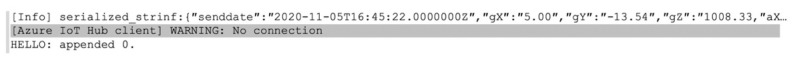
Element added to buffer when no Wi-Fi connection is available.

In order to verify the correct operation of the buffer system implemented on the two boards, the situation where there is no Wi-Fi connection has been simulated. In Figure 11, the Device Output console in Visual Studio reports the moment when an element is appended to buffer.

**Figure 12:**
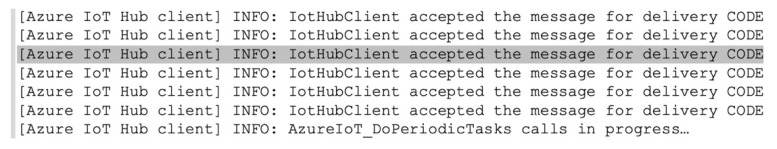
Console showing the buffer emptying when the Wi-Fi connection becomes available again.

When a Wi-Fi connection is available again, the system clears the buffer by sending all data to the IoT Hub, as reported by the Device Output console in Visual Studio in Figure 12.

The experimental results demonstrated that the proposed integrated solution is feasible and effective. Future works will allow to complete with the development of the gray blocks of Figure 5, the implementation of the proposed architecture to obtain a complete, efficient and secure management of the cold chain.

In addition, some machine learning modules (for example, implemented by using the Azure Machine Learning service) could be integrated in the “backend application” to perform predictive analysis (for example, predictive maintenance of containers).

## 7. Discussion

With the proposed solution, we investigated the use of low-cost, secure end devices for fast prototyping developments in the logistics process and specifically in the transportation of goods. In particular, our solution focuses on the integration of Azure Sphere-based hardware, software, and Azure cloud services to support the fast prototyping of solution able to achieve security from the IoT edge devices to the Cloud in the cold chain management. A specific focus has been dedicated to the security. As discussed within the paper, in fact, edge devices are vulnerable to threats and attacks due to the intrinsic low availability of resources. In order to overcome this issue, Azure Sphere certified MT3620 boards have been used as low-cost and high-security monitoring devices in the proposed solution. Azure Sphere and all the services provided by the Azure platform, thanks to their high security and privacy, represent an excellent solution to achieve end-to-end security not only in the logistics field, but also in any Industrial IoT (IIoT) application.

In this regard, it is necessary to discuss the importance of introducing IoT technology into industrial processes, and therefore the importance of ensuring end-to-end security in such processes. Of course, the use of IoT technologies in the industrial field can maximize productivity and reduce costs, while creating new sources of revenue, allowing companies to beat competitors. The following are some examples of the use of IoT technology in the industrial sector and its related benefits:

- Smart supply chain management. IoT technologies make it possible to create smart systems to manage complex supply chains, for example, to obtain demand forecasts and “track-and-trace” monitoring of products to minimize losses.
- Monitoring equipment status and improving production efficiency. By using sensors placed on plant equipment, data can be collected and transmitted to the cloud for analysis and development of predictive maintenance procedures. In this way the company can avoid premature maintenance costs, extend the service life of the machines, and reduce downtime. In addition, the analysis of these data may be useful for the analysis of machinery performance and subsequent adaptation of the production line.
- Optimal facility management. IoT technologies can be used to optimize energy consumption and space usage. In addition, it is possible to create safe conditions for employees and save money due an efficient control of lighting and heating.

All companies that want to take advantage of IoT by using related devices, services and applications should consider security from IoT edge devices to the cloud. In fact, IoT devices are connected to the internet and, therefore, may be attacked by malicious users who control them by stealing data, interrupting service distribution, or performing unreliable operations. These attacks not only cause serious damage to the facilities, but also cause serious damage to the personnel operating or relying on these facilities. In this perspective, Azure Sphere technology can be used to ensure end-to-end security. As the proposed work shows, Azure Sphere MCU, along with its operating system and application platform, enables the creation of secured, internet-connected devices that can be updated, controlled, monitored, and maintained remotely.

Currently, when using an architecture like the proposed rapid prototyping solution, the only negative aspect that can be felt is related to the continuous release of Azure Sphere OS updates, which involves frequent updates of services and related documents. However, it must be specified that, as Azure Sphere is a new technology, such updates are intended to fix bugs or improve functionalities. Moreover, OS updates do not produce costs for a company that uses Azure Sphere solutions. In fact, if the Azure Sphere enabled chip is connected to the Internet, updates to the Azure Sphere OS will be provided directly by providers over-the-air service as part of the Azure Sphere Security Service. In particular, the updates released by Azure during the implementation of the proposed solution did not require any firmware change.

It is worth noting that the proposed solution exploits open source, low-cost tools and hardware. In this way, any company considering adopting a similar architecture can quickly build a prototype solution at low cost and try multiple solutions to verify the most suitable solution for its needs. Using more complex and expensive tools will not be able to get this opportunity. Moreover, the modularity of our solution allows to replace any component with more expensive or less expensive solutions based on specific needs (the replacement of one or more component may require a re-evaluation of the security mechanisms, as end-to-end security may no longer be guaranteed). Finally, with the aim of providing an idea of what “low-cost” stands for, we report here the price at the moment of writing of each component used in the proposed work. The total amount spent for each kit bought to implement the entire system is approximately $80. These kits are intended for rapid prototyping to evaluate the feasibility of a designed solution. However, in order to integrate the hardware certified by Azure Sphere into an actual business environment, the MT3620 MCU could be integrated into existing products/machinery, thereby significantly reducing costs. To understand this concept, it is enough to think that the price of the MT3620 is less than $13, which includes the physical MCU chip, the license of the chip, Azure Sphere OS and Azure Sphere Security Services. On the other hand, with regard to the costs associated with software/cloud components, these costs must be derived from specific business requirements and specific solutions implemented.

In conclusion, Table 9 summarizes the main features that distinguish the proposed work from existing related works. It enriches the discussion with a rapid overview of the main outcome of the presented paper with respect to analyzed state of the art. In details, Table 8 reports information on related works about (a) the use of fast-prototyping solutions throughout the data collection chain, (b) the exploitation of fast-prototyping solutions to ensure security, (c) the adoption of any security measures, (d) the proposal of a modular IoT architecture, and finally (e) the use of modules that are actually exposed to third-party applications, so that researchers and practitioners actually start from them to propose innovative future solutions.

To the best of our knowledge, and as already highlighted in the state-of-the-art analysis, none of existing works supports all the presented features.

## 8. Conclusions

The logistics sector needs to use of innovative IoT technologies to automate, simplify and make its processes more efficient. Different works have already been proposed in literature with the aim of proposing solutions to address such requirements, however, considerations for security and privacy issues need to be deeply investigated paying attention to data from the end device to the cloud. In fact, the IoT ecosystem is composed of many standards, vendors using different hardware, software and third-party services and APIs. This huge fragmentation makes the ecosystem very vulnerable to all sorts of attacks, both at the edge and in the cloud.

In this context, the proposed work exploits an IoT solution entirely based on Microsoft Azure Sphere technologies that is based on the low-cost MT3620 MCU and Microsoft Azure Cloud services to meet the requirement of end-to-end security. With the proposed solution it is possible to exploit fast prototyping tools and technologies to rapidly implement innovative solutions that ensure the highest level of security in products, services and processes in logistics and address the risks and threats at any level. In order to test and verify the effectiveness of the proposed solution, a functional validation has been established on the basis of MT3260 development kit and Azure Cloud services. In the future, other variants of the proposed architecture can be investigated. These may involve the use of hardware that has not been certified by Microsoft and the use of different solutions to ensure end-to-end security. Finally, a real test bed should be configured and implemented that takes into account the use of a multimodal transport system that uses real cargo containers that can be transferred to different modes of transportation (ships, trucks, trains).

## Figures and Tables

**Table 1:** Summary of the main requirements addressed by existing works.

Related Works	Exploit Fast-Prototyping Solutions for Data Acquisition	Exploit Fast-Prototyping Solutions for Security	Security Measures
Chang et al. [24]	No	No	NA
Chen et al. [25]	No	No	NA
Muñoz-Gea et al. [6]	No	No	NA
Qian et al. [7]	No	No	NA
Wanganoo et al. [26]	No	No	NA
Chen et al. [27]	No	No	NA
Zhao et al. [28]	No	No	Yes (custom algorithm)
Belu et al. [29]	No	No	Yes (custom algorithm)
Masood at al. [30]	No	No	Yes (custom algorithm)
Gai et al. [31]	No	No	Yes (blockchain)
Li et al. [32]	No	No	Yes (blockchain)
Madumidha et al. [33]	No	No	Yes (blockchain)
Wrona et al. [22]	Yes	No	Yes (blockchain)
Fedchenkov et al. [23]	Yes	No	No
Azure Sphere platform	Yes	Yes	Yes

**Table 2:** Summary of the first three main edge security requirements addressed by existing works.

Related Works	Exploited MCUs	Hardware-Based Root of Trust	Small Trusted Computing Base	Defense in Depth	Compartmentalization
Chang et al. [24]	8051-like microcontroller unit (MCU)	No	No	No	No
Chen et al. [25]	NA	NA	NA	NA	NA
Muñoz-Gea et al. [6]	NA	No	No	Yes	No
Qian et al. [7]	NA	NA	NA	NA	NA
Wanganoo et al. [26]	NA	NA	NA	NA	NA
Chen et al. [27]	NA	NA	NA	NA	NA
Zhao et al. [28]	NA	NA	NA	NA	NA
Belu et al. [29]	Xilinx Spartan 3 FPGA circuit	Yes	Yes	Yes	No
Masood at al. [30]	NA	NA	NA	NA	NA
Gai et al. [31]	ATmega328P	No	No	No	No
Li et al. [32]	ATmega328P	No	No	No	No
Madumidha et al. [33]	NA	NA	NA	NA	NA
Wrona et al. [22]	NA	Yes	Y/N	Y/N	No
Fedchenkov et al. [23]	NA	NA	NA	NA	NA
Azure Sphere platform	AzureSphere MT3620 MCU	Yes	Yes	Yes	Yes

**Table 3:** Summary of the last four main edge security requirements addressed by existing works.

Related Works	Exploited MCUs	Certificate-Based Authentication	Renewable Security	Failure Reporting	Cost of the MCUs
Chang et al. [24]	8051-like microcontroller unit (MCU)	No	No	No	$1 per piezoelectric
Chen et al. [25]	NA	NA	NA	NA	NA
Muñoz-Gea et al. [6]	NA	Yes	Yes	No	NA
Qian et al. [7]	NA	NA	NA	NA	NA
Wanganoo et al. [26]	NA	NA	NA	NA	NA
Chen et al. [27]	NA	NA	NA	NA	NA
Zhao et al. [28]	NA	NA	NA	NA	NA
Belu et al. [29]	Xilinx Spartan 3 FPGA circuit	No	No	Yes	Xilinx Spartan: $45
Masood at al. [30]	NA	NA	NA	NA	NA
Gai et al. [31]	ATmega328P	No	No	No	Arduino: $30
Li et al. [32]	ATmega328P	No	No	No	Arduino: $30
Madumidha et al. [33]	NA	NA	NA	NA	NA
Wrona et al. [22]	NA	Yes	Yes	Y/N	NA
Fedchenkov et al. [23]	NA	NA	NA	NA	NA
Azure Sphere platform	AzureSphere MT3620 MCU	Yes	Yes	Yes	MT3620: less than $13

**Table 4:** Main technical specifications of Azure Sphere MT3620 kits.

	Seeed Studio Development Kit	AVNET Starter Kit
**MCU**	1 × ARM Cortex A7 core @500 MHz, 4 MB RAM2 × ARM Cortex M4 @200 MHZ, 64 KB RAM	1 × ARM Cortex A7 core @500 MHz, 4 MB RAM2 × ARM Cortex M4 @200 MHZ, 64 KB RAM
**ISU**	4 × ISU serial interfaces which can be configured as: I^2^C runs up to 1 MHz SPI runs up to 40 MHz UART runs up to 3 Mbps	3 × ISU interfaces pre-configured for UART, SPI, I^2^C
**Connectivity**	2.4/5 GHz dual-band 802.11 b/g/n Wi-Fi	Dual-band 2.4/5 GHz 802.11 a/b/g/n Wi-Fi
**ADC**	4 × 12-bit ADC input I/O	ADC/GPIO: 3 × 12-bit ADC inputs (or 3 GPIOs)
**RTC**	1 × RTC with CR2032 3 V battery holder	RTC (requires VBAT supply)
**USB**	1 × Micro USB port for power supply and debugging, 5 V/1 A	1 × USB Interface supports debug, service & recovery UARTs, and JTAG
**Operating Temperature**	−40~85 °C	−40~85 °C
**Dimensions**	L:85 mm × W:50 mm × H:16 mm	
**Certifications**	CE/FCC/MIC/RoHS	FCC/IC/CE/RoHS

**Table 5:** List of commands to manage connection strings.

Command	Meaning
azsphere device wifi list	To display the list of stored Wi-Fi networks
azsphere device wifi show-status	To display the connection status to a network
azsphere device wifi add --ssid <nomerete> --psk <password>	To store a new Wi-Fi network
azsphere device wifi forget --id <id_lista>	To delete a Wi-Fi network

**Table 6:** Main file and related implemented functions.

Board	File Name	Function Name	Meaning
**AVNET**	i2c.c	AccelTimerEventHandler	data detection from sensors check values and send values by calling the “checkSendTelemetry” function
		AzureIoT_SendMessage	sending data in JSON format
		checkSendTelemetry	managing the rules for sending telemetry data to the IoT Hub
	azure_iot_utilities.c	twinCallback	invoked when the DEVICE_TWIN_UPDATE_STATE is saved reads the “desiredProperties” and the “jsonObject sensorsRules” containing the rules for sending the detected values change the sending time, if it has been changed
**SEEED**	main.c	twinCallback	invoked when the DEVICE_TWIN_UPDATE_STATE is saved reads the “desiredProperties” and the “jsonObject sensorsRules” containing the rules for sending the detected values change the sending time, if it has been changed
		checkSendTelemetry	managing the rules for sending telemetry data to the IoT Hub
		SendTelemetryAll	managing the sending rules

**Table 7:** Parameters defined to manage the data transmission from the IoT devices.

Key	Value Type	Option	Description
tv_sec	int	-	time interval, in seconds, for sending messages to the IoT Hub.
<tiporilevazione>	string	temperature|humidity|light|	Sensor type
min	float	-	minimum value to consider in the conditions
max	float	-	maximum value to consider in the conditions
condition	string	in_range|out_range|no_range|gte_min|gte_max|lte_min|lte_m	choice of the control function for sending data
enabled	bool	true|false	defines whether to send the sensor value or not

**Table 8:** Meaning of the options for the “condition” parameter.

Key	Description
in_range	The detected value is sent if it is between the min and max value (including the extremes of the range)
out_range	The detected value is sent if less than/equal to the min value or greater/equal to the max value
no_range	The detected value is sent without any condition
gte_min	The detected value is sent if greater than or equal to the min value
gte_max	The detected value is sent if greater than or equal to the max value
lte_min	The detected value is sent if less than or equal to the min value
lte_max	The detected value is sent if less than or equal to the max value

**Table 9:** Summary of the main features that distinguish the proposed work from existing related works.

Related Works	Exploit Fast-Prototyping Solutions for Data Acquisition	Exploit Fast-Prototyping Solutions for Security	Security Measures	Modular IoT Architecture	Used Modules are not Exposed to Third Parties
Chang et al. [24]	No	No	NA	No	NA
Chen et al. [25]	No	No	NA	Yes	No
Muñoz-Gea et al. [6]	No	No	NA	Yes	Not all
Qian et al. [7]	No	No	NA	NA	NA
Wanganoo et al. [26]	No	No	NA	Yes	No
Chen et al. [27]	No	No	NA	Yes	No
Zhao et al. [28]	No	No	Yes (custom algorithm)	Yes	No
Belu et al. [29]	No	No	Yes (custom algorithm)	No	NA
Masood at al. [30]	No	No	Yes (custom algorithm)	No	NA
Gai et al. [31]	No	No	Yes (blockchain)	No	NA
Li et al. [32]	No	No	Yes (blockchain)	No	NA
Madumidha et al. [33]	No	No	Yes (blockchain)	No	NA
Wrona et al. [22]	Yes	No	Yes (blockchain)	Yes	Yes
Fedchenkov et al. [23]	Yes	No	No	Yes	Yes
Proposed system	Yes	Yes	Yes	Yes	Yes

## Data Availability

Data sharing not applicable.
